# Light Absorption Enhancement of Silicon-Based Photovoltaic Devices with Multiple Bandgap Structures of Porous Silicon

**DOI:** 10.3390/ma8095283

**Published:** 2015-09-07

**Authors:** Kuen-Hsien Wu, Chong-Wei Li

**Affiliations:** Department of Electro-Optical Engineering, Southern Taiwan University of Science and Technology, No. 1, Nan-Tai Street, Yungkang Dist., Tainan 710, Taiwan; E-Mail: 499l0100@stust.edu.tw

**Keywords:** porous silicon, multi-layer, multi-bandgap, photoresponse, photovoltaic device, solar cell

## Abstract

Porous-silicon (PS) multi-layered structures with three stacked PS layers of different porosity were prepared on silicon (Si) substrates by successively tuning the electrochemical-etching parameters in an anodization process. The three PS layers have different optical bandgap energy and construct a triple-layered PS (TLPS) structure with multiple bandgap energy. Photovoltaic devices were fabricated by depositing aluminum electrodes of Schottky contacts on the surfaces of the developed TLPS structures. The TLPS-based devices exhibit broadband photoresponses within the spectrum of the solar irradiation and get high photocurrent for the incident light of a tungsten lamp. The improved spectral responses of devices are owing to the multi-bandgap structures of TLPS, which are designed with a layered configuration analog to a tandem cell for absorbing a wider energy range of the incidental sun light. The large photocurrent is mainly ascribed to an enhanced light-absorption ability as a result of applying nanoporous-Si thin films as the surface layers to absorb the short-wavelength light and to improve the Schottky contacts of devices. Experimental results reveal that the multi-bandgap PS structures produced from electrochemical-etching of Si wafers are potentially promising for development of highly efficient Si-based solar cells.

## 1. Introduction

Since the report of room temperature photoluminescence in porous silicon (PS) [1], this material has been extensively investigated for the development of optoelectronic devices. PS is constituted by a nanocrystalline skeleton (quantum sponge) immersed in a network of pores and can be obtained simply by electrochemical etching of silicon in aqueous hydrofluoric (HF) [2,3]. Due to its many special characteristics such as extremely large surface-area-to-volume ratio, modulated energy bandgap, absorption spectrum within the solar irradiation, surface roughening with low reflection losses, and high photoconductivity, PS is very suitable for applications in photovoltaic devices [4]. In particular, PS structures have been intensively introduced in the device design of solar cells to increase the cell’s conversion efficiency [5,6,7]. In most of the related research, PS films acted as the surface texturing and anti-reflection (AR) layers to reduce the light-reflection losses or as the surface passivation layers to decrease surface carrier recombination velocity. On the other hand, PS can potentially serve as the parts of the active layer to effectively generate photo-induced carriers because of its wide energy bandgap and high photoconduction in response to solar irradiation [8,9]. However, a primary drawback of PS for use of the light-absorption layer in a photovoltaic device is its comparatively narrow spectral photoresponses. Therefore, to get higher and broader spectral photoresponses within the spectrum of the solar irradiation is a primary requirement for the fabrication of efficient PS-based solar cells.

An important scheme to increase the light absorbance range of devices is using cell structures with multi-junctions (*i.e.*, the “tandem cell” structures). Tandem cells are solar cells with multiple *p–n* junctions made of different semiconductor materials with different bandgap energy. Because each semiconductor material is sensitive to a different part of the solar spectrum, tandem cells can obtain enhanced overall sunlight photoresponses and achieve higher conversion efficiency. However, these tandem cells are usually not suitable for mass production of silicon (Si) solar cells since their fabrication processes are so complicated and are not Si-compatible [10,11]. 

A promising Si-based tandem-like structure is the PS multilayer (PSML) structure composed of stacked PS layers with different porosity. In the past, PSML had received much interest due to its tunable optical and electronic properties [4]. Because the refractive index of PS can be tuned through the porosity using controlled electrochemical-etching conditions, PSML with modulated refractive index can be fabricated by stacking PS layers of different porosity. These PSML structures can be used to make optical filters, waveguides, resonating cavities and mirrors for device applications [12,13]. Most of the recent studies about applications of PSML in solar cells mainly focused at the optimization of AR coatings [14,15]. There are few reports on cells with light-absorption areas based on PSML structures. Since the optical bandgap of PS can be tuned by its porosity, it is feasible to broaden the photoresponse spectra of Si photovoltaic devices by use of multi-bandgap PSML structures. Based on the fact that a PS layer of higher porosity has higher bandgap energy, a PSML structure composed of triple stacking layers with high-, medium- and low-porosity (from top to bottom) will absorb the sun light in the short-, medium- and long-wavelength bands respectively. Such a “tandem-like” PSML structure can potentially harvest a broader range of the sun’s energy to make a Si-based solar cell more efficient. 

Utilization of PSML as the light-absorption region of a device will face a dilemma in choosing the porosity of the upmost PS layer. To absorb light of shorter wavelength, the upmost PS layer must be designed with higher porosity. However, a PS layer with higher porosity has higher resistance, lower hardness and higher density of defects [4,5]. Therefore, using a high-porosity PS surface layer will result in high series resistance and degradation of metal contacts. To solve this problem, we proposed a special nanoporous-Si (NPS) thin film for serving as the surface active layer. The NPS layer associated with two stacked PS layers of different porosity will construct a triple-layered PS (TLPS) structure with multiple bandgap energy. Such a TLPS structure can be produced on Si substrates by tuning the etching parameters in an electrochemical anodization process. In this paper, we report the preparation and characterization of TLPS structures and demonstrated the improvements on the spectral photoresponses and light-absorption capability of Si-based photovoltaic devices fabricated with the developed TLPS structures.

## 2. Experimental Section

### 2.1. Formation of Triple-Layered Porous Silicon

N^+^-type (100) silicon wafers with resistivity of 5–10 mΩ·cm were used as the starting substrates. PS layers were prepared on Si substrates by electrochemical etching in an anodization process with HF-methanol etching solutions in a Teflon cell with a Pt electrode. To generate a TLPS structure, the electrochemical-etching parameters were successively changed in the anodization process. Different HF concentration of etching solution, etching current density and etching time were used for formation of different PS stacked layers with varied morphology and porosity. Table 1 lists the etching parameters used in the anodization process for preparation of the triple stacked PS layers. To obtain a NPS thin film as the upmost (the first) layer, lower etching current density of 10 mA/cm^2^ was used during the anodic process. The HF concentration of the etching solution is the key parameter to control the sizes and distribution of Si-nanocrystals in the NPS films. In order to get smaller sizes of Si-nanocrystals and higher reproducibility, the concentration of HF was set as 30% of the etching solution in this work.

**Table 1:** Electrochemical-etching parameters used in the anodization process for preparation of the triple stacked PS layers.

Etching Parameters	First Layer (Upmost)	Second Layer (Middle)	Third Layer (Bottom)
HF Concentration	30%	10%	10%
Etching Current Density (mA/cm^2^)	10	60	30
Etching Time (s)	60	60	90

To create the following PS layers, HF concentration of the etching solution was reduced *insitu* to 10% and the etching current density was increased to 60 and 30 mA/cm^2^ for formation of a high-porosity PS (HP-PS) layer as the second layer and a low-porosity PS (LP-PS) layer as the third layer, respectively. Because etching only occurs at the PS-Si interface, the previously formed PS layer will be left unaffected. After the formation of PS layers, samples with the prepared TLPS were rapid-thermally annealed in N_2_ at 800 °C for 60 s to stabilize the PS structures. 

**Figure 1:**
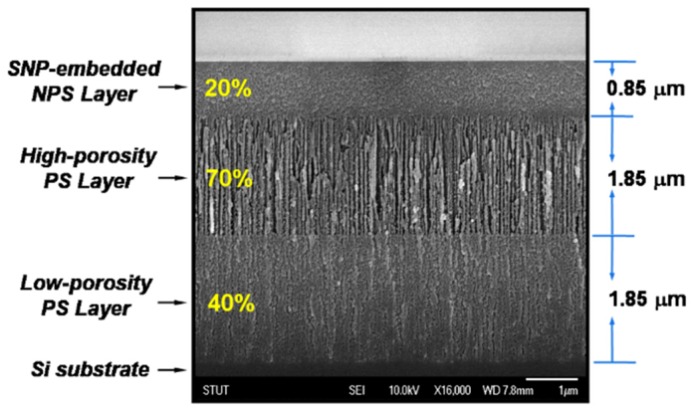
The SEM photo-image of the cross-sectional view of the formed triple-layered porous-silicon (TLPS) structure on a Si substrate. The triple layers include a nanoporous-Si (NPS), a high-porosity PS and a low-porosity PS layer from top to bottom.

Figure 1 illustrates the scanning-electron-microscope (SEM) photo-image of the cross-sectional view of the produced TLPS structure on a Si substrate. It can be observed that a triple-layered structure with an NPS, an HP-PS and a LP-PS layer stacking from top to bottom are formed on a Si substrate. The thickness of each layer is about 0.85 μm, 1.85 μm and 1.85 μm, respectively. The upmost NPS layer has a different morphological structure from that of the HP-PS or the LP-PS layer. The NPS layer is a particulate thin film consisting of uniformly distributed Si-nanoparticles (SNP’s), while the HP-PS and the LP-PS layers are with structures containing pipe-shaped micro-rods and micro-holes. For determining the porosity of the three intermediate PS layers, individual PS layer was also prepared by use of the same etching parameters listed in Table 1. The porosity of the NPS, HP-PS and LP-PS layer is estimated by weight measurements to be about 20%, 70% and 40%, respectively.

### 2.2. Fabrication of Photovoltaic Devices

**Figure 2:**
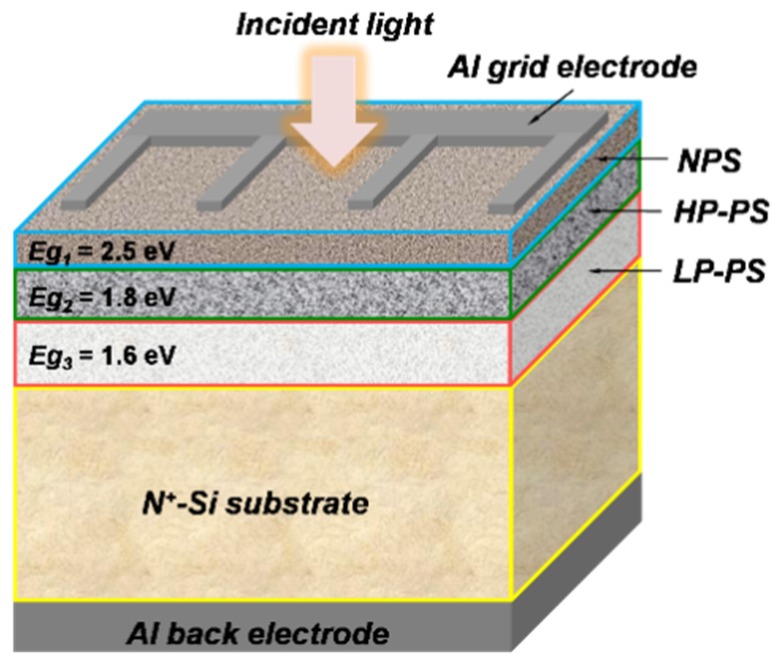
Schematic structure of the developed Si-based photovoltaic device with a triple-layered porous-Si (TLPS) structure as the light-absorption region.

After the preparation of TLPS, aluminum (Al) was deposited on the back side of the sample and then sintered at 350°C to make the back electrode of Ohmic contact. Thereafter, grid Al metals were formed on the front side of the sample to serve as the front electrodes of Schottky contact. Figure 2 shows the schematic structure of the developed Si-based photovoltaic device with the TLPS serving as the main light-absorption region. By means of the Tauc’s plots [16], the optical bandgap energy (*Eg_opt_*) of the individual as-prepared PS layer is estimated to be 2.5 eV, 1.8 eV, and 1.6 eV for the NPS, the HP-PS, and the LP-PS layer, respectively. Like a tandem cell, the developed device has a light-absorption region with multiple optical bandgaps and the bandgap of each layer in the light-absorption zone decreases from the device’s surface to its interior.

## 3. Results and Discussion

**Figure 3:**
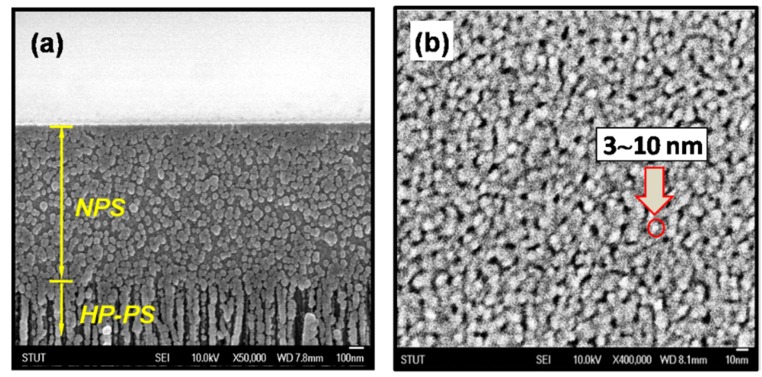
(**a**) The zoom-in SEM photo-image of the prepared NPS layer shown in Figure 1; (**b**) A high-magnification SEM image of the surface of the prepared NPS film.

Figure 3a shows the zoom-in SEM photo-image of the prepared NPS layer in Figure 1. It can be observed that the NPS layer has a special film-like structure, unlike the beneath HP-PS layer which has the common morphology of meso- or micro-PS layers generated from electrochemical-etching of Si wafers. Figure 3b shows a high-magnification SEM image of the surface of the NPS film. As shown in this figure, the NPS film is embedded with uniformly distributed Si-nanocrystals and nano-sized holes. Average sizes of these Si-nanocrystals are estimated to be about 3–10 nm in diameters. According to the reports of Lee *et al.* [17], the bandgap energy of a PS material with quantum-sized Si-crystallites of 3 nm is about 2.5 eV. This value is close to that was predicted by Suemune *et al.* [18] and conforms to the *Eg_opt_* value of the NPS film measured from the Tauc’s plots in this work. The *Eg_opt_* of the beneath HP-PS layer with porosity of 70% and the LP-PS layer with porosity of 30% got from the Tauc’s plots is about 1.8 eV and 1.6 eV, respectively. These values are consistent with those obtained from the optical absorption measurements in other studies [17].

**Figure 4:**
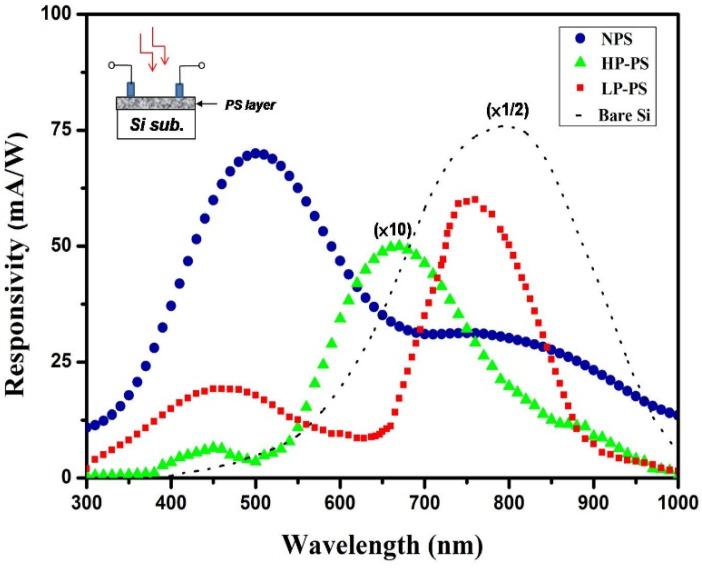
The spectral photoresponses of the individual PS (NPS, HP-PS or LP-PS) layer prepared on a Si substrate, measured with planar electrodes on the PS surface as indicated in the inserted drawing. For comparison purposes, the measured data of the bare Si substrate are also shown.

Figure 4 shows the spectral photoresponses of the individual PS (including NPS, HP-PS and LP-PS) layer prepared on a Si substrate. The photoresponses were measured with a TRIAX-320 spectrometer (HORIBA, Kyoto, Japan). To exclude as much as possible the influence of light absorption in the Si substrate, the responsivity was measured by using planar electrodes formed on the surface of the PS layer, as indicated in the inserted drawing. For comparison purposes, photoresponses of the bare Si substrate are also shown in the figure. As shown in the figure, the photoresponses of the LP-PS layer mainly appear within the ranges of wavelength between 650 nm and 850 nm, which corresponds to the optical band from red to infra-red (IR), and has a maximum responsivity of 62 mA/cm^2^ at 750 nm. The LP-PS layer displays the common photoresponsive characteristics of a PS material, which has a narrower and blue-shifted response spectrum with smaller responsivity as compared to that of the bare Si. The responses of the HP-PS device shift further toward the band of shorter-wavelength between 550 nm (green) and 700 nm (red) with a much small peak responsivity of 5 mA/cm^2^ at 670 nm. We thought the change in the region of the spectral responses is due to the higher porosity of layers which result in a larger *Eg_opt_*. The low responsivity is ascribed to not only its high resistivity and the high defect density, but also the large amount of the surface states and the high metal-contact resistance. By improving the metal contacts and the surface passivation on the PS layer, the responsivity of high-porosity PS can be largely enhanced [19,20]. Particularly in this work, the HP-PS layer was made under the NPS surface layer in the fabricated TLPS-based device. As a result, the surface of the HP-PS is effectively passivated by the NPS layer and the quality of metal-contact is thus not degraded by the HP-PS layer since the electrodes are formed on the NPS layer that has a surface with better quality. Therefore, the actual photoresponsivity of such a middle HP-PS layer in the TLPS structure is expected to be higher than that of an individual HP-PS layer as a surface layer on a Si substrate.

As shown in Figure 4, the NPS layer not only exhibits much higher short-wavelength responses within 400–600 nm in the blue band (with a maximum responsivity of 72 mA/cm^2^ at 500 nm), but also has apparent responses in the band of red-to-IR. It is supposed that the greatly enhanced blue-shifted responses come from the SNP’s embedded in the NPS films, which has *Eg_opt_* larger than 2 eV due to the quantum-size effects. The red-to-IR responses are ascribed to the light absorption in the micro-sized Si crystals around the SNP’s and the beneath Si substrate. Besides, it is interesting to find that the value of the peak responsivity of the NPS, HP-PS, and LP-PS layer corresponds respectively to a photon energy of 2.48 eV, 1.77 eV, and 1.65 eV, which is close to the value of *Eg_opt_* of the single PS layer respectively. This fact indicates that the *Eg_opt_* of a PS layer has significant influences on its photoresponses.

**Figure 5:**
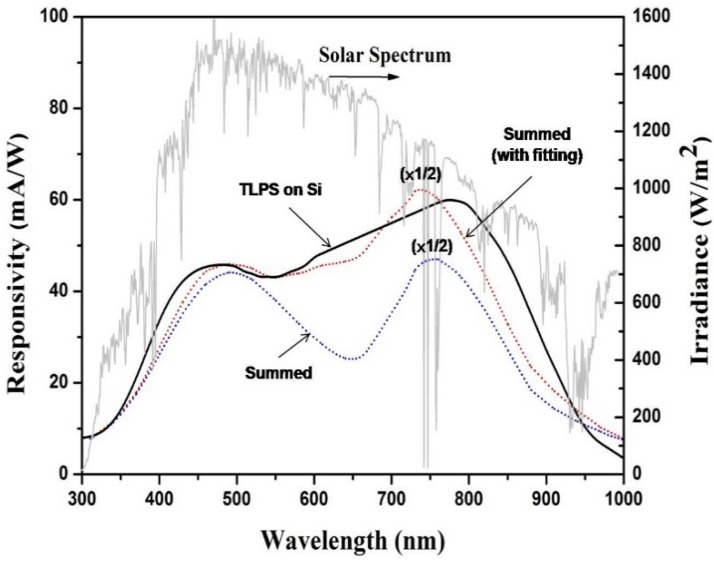
The spectral photoresponses of the developed photovoltaic devices based on the TLPS structure and the individual PS (NPS, HP-PS or LP-PS) layer. The summed data (blue dotted line) were obtained by directly summing the three individual responses of the PS layer in Figure 4, while the summed (with fitting) data (red dotted line) were obtained by using a 10-fold responses of HP-PS. The spectrum of solar irradiation is also shown in the figure for comparison purposes.

Figure 5 illustrates the spectral photoresponses of the photovoltaic devices with a TLPS structure on a Si substrate.

It is obvious that the TLPS-based device exhibits a broader photoresponse spectrum than that of an individual PS layer and the bare Si substrate. Most importantly, the developed device achieved a broadened and flat photoresponse spectrum that matches much more closely to that of the solar irradiance. That is a preferable property for development of highly efficient solar cell. From the spectral distribution of photoresponses of the three individual PS layer in Figure 4, it is reasonable to suppose that the overall responses of a TLPS device are primarily composed of the generated responses in the three PS layers of a TLPS structure. To verify this assumption, we plotted the summed responses (denoted as “Summed”) that were obtained by directly summing the three individual responses of the PS layer in Figure 4. The shown responses from the summed data were scaled down twofold for curve fitting, based on the fact that the resistance of the individual PS layer is smaller than that of a TLPS. It is apparent that the summed response spectra bend down between 550 nm and 750 nm as a result of the relatively small values of the individual HP-PS layer. However, the HP-PS is expected to exhibit higher responses as a middle layer in the TLPS structure due to the passivation of the NPS layer. By using a 10-fold response of HP-PS for summing, the plotted summed response spectra (denoted as “Summed (with fitting)”) become much flatter and more fitting to that of a TLPS-based device. These results support the expectation that the middle HP-PS layer can generate higher photoresponses than those of a surface HP-PS layer and indicate the assumption that the measured wide-band photoresponses come from a synergistic function of the three PS layers in the TLPS structure is valid.

**Figure 6:**
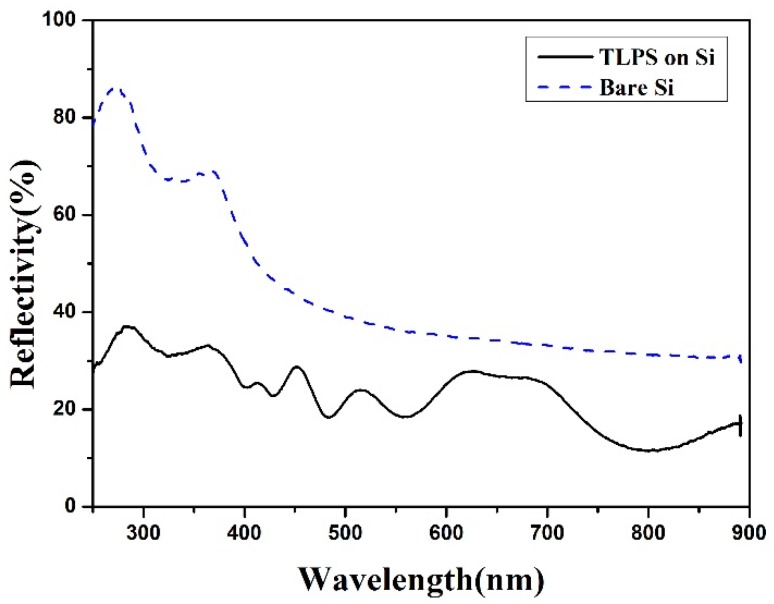
The spectral reflectance of the TLPS structure on a Si substrate and the bare Si substrate.

Furthermore, to check whether the multilayered PS structure on top of the Si substrate causes an interference effect on the photoresponses of the TLPS device, the spectral reflectance of the TLPS on a Si substrate was measured and shown in Figure 6. From this figure, we can find that the average reflectivity of samples with TLPS on Si is about two times lower than that of the bare Si substrate in the visible band. That verifies the TLPS structure enhanced the light absorption. There are no obvious interference patterns appearing in the spectral reflectance. It indicates that the layered structure of TLPS in this work has a negligible interference effect on the generation of the spectral responses. Therefore, the improved spectral responses of the TLPS-based device are mainly owing to the multi-bandgap TLPS structures in which different layers with different *Eg_opt_* absorb different portions of the incident light and contribute all together to the whole photoresponses.

In a tandem cell, layers with different bandgap are stacked on top of one another with the highest bandgap layer upmost. Since layers with higher bandgap will absorb light of shorter wavelength, the device’s conversion efficiency can be largely increased if bandgap energy of each layer is optimally chosen. According to related researches, light-absorption spectra of PS with higher porosity usually shift towards shorter wavelength because of the larger bandgap energy of PS [21]. These results accorded with the experimental ones in this work. In other words, PS layers with larger bandgap energy would absorb light of shorter wavelength. For the developed TLPS devices, the *Eg_opt_* value of each layer (from surface to the interior) in the light-absorption zone was 2.5 eV (NPS), 1.8 eV (HP-PS), 1.6 eV (LP-PS) and 1.1 eV (bulk Si), respectively. Since these *Eg_opt_* values spanned the main ranges of photon energy in the solar spectrum, the TLPS devices exhibited broadband photoresponses and were able to harvest more solar energy.

**Figure 7:**
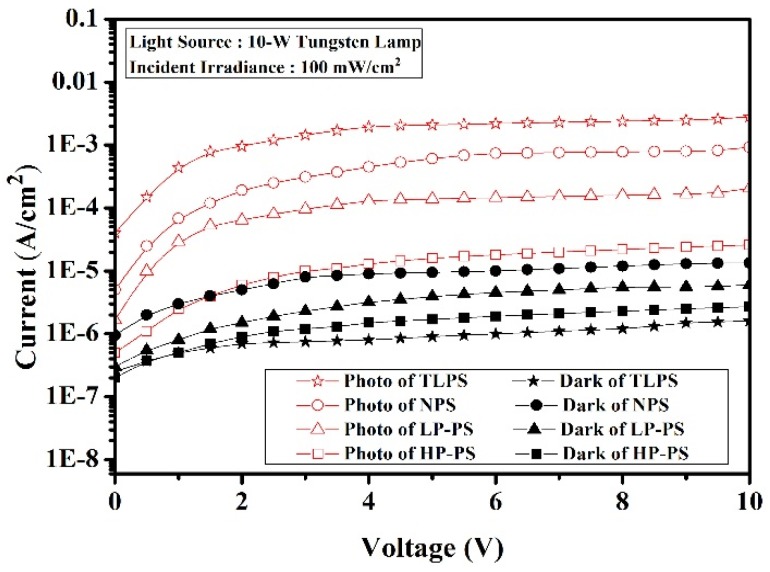
Photo and dark current-voltage (I–V) characteristics of photovoltaic devices based on the TLPS structures and the individual PS (NPS, LP-PS or HP-PS) layer on a Si substrate.

To assess the influences of the improved photoresponses on the performance of the photovoltaic devices, photo- and dark current-voltage characteristics of a TLPS-based device were measured and plotted in Figure 7. For comparison purposes, similar devices based on a single PS (NPS, LP-PS or HP-PS) layer with a comparable thickness of about 4.5 μm were also fabricated and measured. The photocurrent of devices was measured under an incident irradiation of 100 mW/cm^2^ by using a 10-W tungsten lamp with emitting light of wavelength ranging from 300 nm to 1100 nm and a peak wavelength of 550 nm. The reason for choosing such a tungsten lamp as the light source is that it has an irradiation spectrum similar to that of the sun. As observed in the figure, the TLPS-based device obtains the largest photocurrent and smallest dark current among these fabricated devices, indicating it has the most significant photoconduction property. Because the photoresponse spectrum of a TLPS device matched much more closely to that of the incident light, the higher photocurrent is mainly attributed to its better light-absorption ability coming from its improved photoresponses. The increase in the photocurrent indicates higher short-circuit current and higher conversion efficiency will be obtained by application of the TLPS structures. At a reverse bias of 5 V, the TLPS device has about one-order higher photocurrent than that of one based on a single LP-PS layer with porosity of 40%, which was applied as the surface film in a Si-based solar cell to achieve optimum device performance [8]. That imply that the TLPS structures have good potential to improve the performance of a Si-based photovoltaic device. 

The photocurrent of an NPS-based device is about half of that of the TLPS one at 5 V. From this data and the photoresponses of the individual NPS layer shown in Figure 4, we deduced that the NPS layer contributes the most part of the photo-induced current to a TLPS device. In fact, there are many advantages by incorporating the NPS layer as the surface layer in the device. First, the NPS layer significantly increases the responses in the short-wavelength ranges around 450–550 nm that are just the portions including the maximum irradiance of the incident light (or the sun light), as we can observed in Figure 4 and Figure 5. Second, the micro-holes in the NPS layer provide a textured surface structure to reduce the light-reflection losses and augment the light absorption of devices. Third, as mentioned above, the NPS layer effectively passivates the lower HP-PS layer for complementing the middle-band photorespnses. Four, the smooth and dense surface of the NPS layer ensures the formation of a Schottky contact with high quality between the metal electrodes and the PS layer. This advantage can be realized from the comparison of the measured dark current between the TLPS and the HP-PS devices. Although a HP-PS device has larger series resistance than that of a TLPS device, it has higher dark current as shown in Figure 7. For a Schottky device, high density of defects at the metal-semiconductor (M-S) interface will reduce the Schottky barrier of the M-S junction, thus causing a decrease in the photocurrent and increase in the reverse (or leakage) current [22]. In a photovoltaic device, the leakage current dominates the dark current. That is, a PS-based photovoltaic device with a surface PS layer of higher-porosity usually had lower photocurrent and higher dark current due to the degraded Schottky contact. Therefore, the large amount of defects on the surface HP-PS layer cause high dark current. On the contrary, due to the incorporation of an NPS surface layer, the TLPS device with a middle HP-PS layer has a good Schottky contact to generate a higher photocurrent and retain lower dark current. Since the photo-to-dark current ratio (PDCR) is an important figure of merit for the performance of a photovoltaic device, the large PDCR of the fabricated TLPS-based device demonstrates that the multi-bandgapped PS structure has high potential in development of Si-based photovoltaic device.

## 4. Conclusions

By successively altering the etching parameters in an electrochemical anodization process, TLPS structures with NPS/HP-PS/LP-PS stacked layers can be produced on Si substrates. The three stacked PS layers in the formed TLPS structure have varied optical bandgap energy decreasing from top to bottom and provide the TLPS structure with multiple bandgaps. Experimental results show that the fabricated photovoltaic devices based on TLPS structures exhibit broadband photoresponses within the spectrum of the solar irradiation and get high photocurrent for the incident light of a tungsten lamp. The improved spectral responses are ascribed to the multi-bandgap structures of TLPS in which the separate PS layer with distinct optical bandgap energy absorbs different energy portions of the incident light and contributes all together to the total photoresponses. Most notably, the use of NPS films as the surface layers greatly enhances the light-absorption ability of devices by supplying greatly increased short-wavelength responses and providing good Schottky contacts to get large photocurrents and reduce the leakage current. Therefore, the developed TLPS structures have high potential in fabrication of Si-based solar cells with enhanced conversion efficiency.
